# Deciphering the molecular effects of romidepsin on germ cell tumours: DHRS2 is involved in cell cycle arrest but not apoptosis or induction of romidepsin effectors

**DOI:** 10.1111/jcmm.13971

**Published:** 2018-11-20

**Authors:** Daniel Nettersheim, Daniel Berger, Sina Jostes, Margaretha Skowron, Hubert Schorle

**Affiliations:** ^1^ Department of Urology, Urological Research Lab, Translational Urooncology University Medical School Düsseldorf Düsseldorf Germany; ^2^ Department of Developmental Pathology Institute of Pathology, University Medical School Bonn Bonn Germany

**Keywords:** DHRS2/HEP27, epigenetic therapy, germ cell tumour, histone deacetylase inhibitor, romidepsin

## Abstract

Testicular germ cell tumours (GCTs) mostly affect young men at age 17‐40. Although high cure rates can be achieved by orchiectomy and chemotherapy, GCTs can still be a lethal threat to young patients with metastases or therapy resistance. Thus, alternative treatment options are needed. Based on studies utilising GCT cell lines, the histone deacetylase inhibitor romidepsin is a promising therapeutic option, showing high toxicity at very low doses towards cisplatin‐resistant GCT cells, but not fibroblasts or Sertoli cells. In this study, we extended our analysis of the molecular effects of romidepsin to deepen our understanding of the underlying mechanisms. Patients will benefit from these analyses, since detailed knowledge of the romidepsin effects allows for a better risk and side‐effect assessment. We screened for changes in histone acetylation of specific lysine residues and analysed changes in the DNA methylation landscape after romidepsin treatment of the GCT cell lines TCam‐2, 2102EP, NCCIT and JAR, while human fibroblasts were used as controls. In addition, we focused on the role of the dehydrogenase/reductase *DHRS2*, which was strongly up‐regulated in romidepsin treated cells, by generating *DHRS2*‐deficient TCam‐2 cells using CRISPR/Cas9 gene editing. We show that DHRS2 is dispensable for up‐regulation of romidepsin effectors (*GADD45B*,*DUSP1*,*ZFP36*,*ATF3*,*FOS*,*CDKN1A*,*ID2*) but contributes to induction of cell cycle arrest. Finally, we show that a combinatory treatment of romidepsin plus the gluccocorticoid dexamethasone further boosts expression of the romidepsin effectors and reduces viability of GCT cells more strongly than under single agent treatment. Thus, romidepsin and dexamethasone might represent a new combinatorial approach for treatment of GCT.

## 1 INTRODUCTION

Testicular type II germ cell tumours (GCTs), which are sub‐divided into seminomas and non‐seminomas, arise from the precursor lesion germ cell neoplasia in situ (GCNIS).^1^, ^2^ GCNIS and seminoma cells are highly similar to primordial germ cells with regard to histology, gene expression and epigenetics, while the stem cell population of the non‐seminomas, the embryonal carcinoma (EC), shows features of pluri‐ to totipotency.^1^ Thus, ECs are able to differentiate into cells of all germ layers (teratoma) and into cells resembling extra‐embryonic tissues (yolk‐sac tumours, choriocarcinomas).^1^

Generally, GCTs are treated by orchiectomy followed by additional chemotherapy. By this, high cure rates of >90% are achieved, however, patients with metastatic disease or resistance towards standard chemotherapy require alternative therapeutic options.^3^

In previous studies, we demonstrated that treatment of (cisplatin‐resistant) GCT cell lines with the histone deacetylase inhibitor (HDACi) romidepsin (ISTODAX, FR228, FR901228) efficiently induced apoptosis and blocked the cell cycle at very low doses, but did not affect survival of fibroblasts or Sertoli cells.^4^, ^5^, ^6^ We showed that romidepsin treatment of GCT cell lines resulted in heterochromatin formation within the promotor of *ARID1A* causing down‐regulation of *ARID1A,* 5 which is a subunit of the chromatin remodelling SWI/SNF‐complex. As a result, stress and apoptosis sensors as well as cell cycle regulators *GADD45B, ATF3, ZFP36, DUSP1, FOS, ID2* and *CDKN1A* were up‐regulated.^5^ In addition, we identified four genes (*DHRS2*,* RHOB*,* CRISPLD2*,* BAIAP2*), which were up‐regulated in all GCT cell lines tested as well as the controls (fibroblasts and the Sertoli cell line FS1), suggesting that these genes represent a common effect of romidepsin on gene expression.^5^ Among all samples analysed, *DHRS2* was the most prominently up‐regulated gene.^5^

In this study, we extended our analysis of the molecular mode of action of romidepsin and also focused on the role of *DHRS2*, a NADPH‐dependent dehydrogenase/reductase, in the romidepsin‐response cascade, to gain a better understanding of the effects of romidepsin on GCTs and normal healthy cells. In general, understanding the molecular effects of a therapeutic drug is required for the assessment of risks and side effects before administering it to a patient.

## 2 MATERIALS AND METHODS

### 2.1 Cell culture

All cell lines used as GCT proxies (TCam‐2, seminoma; 2102EP, EC; NCCIT; extra‐gonadal derived EC; JAR, placenta‐derived choriocarcinoma), fibroblasts (MPAF) and Sertoli cells (FS1) used in this study were cultivated as described previously.^5^ MPAF were provided by Dr. Michael Peitz (Life & Brain, Department of Reconstructive Neurobiology, Bonn, Germany) and FS1 were provided by Dr. Valerie Schumacher (Boston Children's Hospital, Boston, MA).^7^

### 2.2 HDACi and dexamethasone application

Romidepsin was dissolved and applied as described before.^5^ Romidepsin was provided by Gloucester Pharmaceuticals (Celgene; Signal Pharmaceuticals, LLC, San Diego, CA; MTA ID #CC0488464). Dexamethasone (Sigma‐Aldrich, Taufkirchen, Germany) was dissolved in 100% ethanol to 1 mg/mL.

### 2.3 Generation of *DHRS2*‐deficient TCam‐2 cells

TCam‐2 cells heterozygous or homozygous deficient for *DHRS2* were generated as published.^5^, ^8^ Deletions within the coding sequence of *DHRS2* in each clone were detected by PCR (Figure S1C,D). See Table 1 for guideRNA sequences and genotyping primers.

**Table 1:** Oligonucleotides used in this study

Gene	Forward primer	Reverse primer	Tan	Cycles
ARID1A	TCTTGCCCATCTGATCCATT	CCAACAAAGGAGCCACCAC	60°C	40
ATF3	AAGAACGAGAAGCAGCATTTGAT	TTCTGAGCCCGGACAATACAC	60°C	40
CDKN1A	CCTCATCCCGTGTTCTCCTTT	GTACCACCCAGCGGACAAGT	60°C	40
DHRS2	CTCCATGTAGGGCAGCAACT	GTAGGGAGCACTCTGGGGAC	60°C	40
DHRS2 genotyp. primer pair 1	GGAAGGACAGTGGAGAGAGG	CCGACTGTATTTCTGTGCCC	60°C	35
DHRS2 genotyp. Primer pair 2	AGAGCTGGGTAGAGGAAGGA	TACAGGCACAGGTCACCAAA	55°C	35
DUSP1	GTACATCAAGTCCATCTGAC	GGTTCTTCTAGGAGTAGACA	60°C	40
FGF13	TGAATTTGCACTCAGGTGTGA	GTCTGCGAGTGGTGGCTATC	60°C	40
FOS	GAGAGCTGGTAGTTAGTAGCATGTTGA	AATTCCAATAATGAACCCAATAGATTAGTTA	60°C	40
GADD45B	GTCGGCCAAGTTGATGAAT	CACGATGTTGATGTCGTTGT	60°C	40
GAPDH	TGCCAAATATGATGACATCAAGAA	GGAGTGGGTGTCGCTGTTG	60°C	40
ID2	TCAGCCTGCATCACCAGAGA	CTGCAAGGACAGGATGCTGATA	60°C	40
MYC	CGTCTCCACACATCAGCACAA	CACTGTCCAACTTGACCCTCTTG	60°C	40
P53	TTGCAATAGGTGTGCGTCAGA	AGTGCAGGCCAACTTGTTCAG	60°C	40
RHOB	GGGACAGAAGTGCTTCACCT	CGACGTCATTCTCATGTGCT	60°C	40
TUFT1	CCTGTCAGTTCACCCTGGAG	AACTGGTGTACCCTGGTGGA	60°C	40
VAMP1	CAGTCCCTTCTGTCCCTTCA	CAGCCTCCGGAGAGGAA	60°C	40
ZMYND11	TTGTTAAACGTGCCATGACC	GCATGTGTGGAGACAGAGGA	60°C	40

Gene	gRNA sequence
DHRS2 gRNA 1	5′‐CTTTGCCATCGCCCGACGTC‐3′
DHRS2 gRNA 2	5′‐GCCATACCTGTTCTCCATGT‐3′
DHRS2 gRNA 3	5′‐CCCAGAGTGCTCCCTACCAG‐3′

### 2.4 DNA, RNA and protein isolation

Genomic DNA, total RNA and proteins were isolated as described previously.^5^ Briefly, DNA was isolated by phenol/chloroform/isoamylalcohol precipitation, RNA by the RNAeasy mini kit (Qiagen, Hilden, Germany) and proteins by RIPA buffer.

### 2.5 Western blot

Western blots were performed as described previously.^5^ Beta‐ACTIN was used as housekeeper and loading control. See Table 2 for antibody details.

**Table 2:** Antibodies used in this study

Antibody	Company	Clone/order no.	Western blot
Beta‐Actin	Sigma‐Aldrich	AC‐15	1:20 000
GR	Abcam	ab3671	1:400
H3 pan‐ac	Active motif	39139	1:500
H4 pan‐ac	Active motif	39243	1:750
H3K4ac	Active motif	39381	1:2000
H3K9	Active motif	61251	1:2000
H3K14ac	Active motif	39599	1:2000
H3K18ac	Active motif	39587	1:2000
H3K23ac	Active motif	39131	1:2000
H3K27ac	Active motif	39685	1:2000
H3K36ac	Active motif	39379	1:2000
H3K37ac	Active motif	61587	1:2000
H3K56ac	Active motif	39281	1:2000
H3K79ac	Active motif	39565	1:2000
H4K5ac	Active motif	61523	1:1500
H4K8ac	Active motif	39171	1:1500
H4K12ac	Active motif	39165	1:1500
H4K16ac	Active motif	39167	1:1500
H4K20ac	Active motif	61531	1:1500

### 2.6 Quantitative RT‐PCR

Quantitative RT‐PCR was performed as published previously.^5^ 500 ng of total RNA was used for first strand synthesis. *GAPDH* was used as housekeeping gene and for data normalisation. In general, all samples were analysed in technical triplicates and biological triplicates/quadruplicates (see individual figure legend for more detailed information).

### 2.7 Quantification of DNA methylation levels

DNA methylation (5mC) levels were quantified as published using the “MethylFlash Methylated DNA 5‐mC Quantification Kit (Colorimetric)” (Epigentek, via BioCat, Heidelberg, Germany).^12^ 200 ng of genomic DNA was used. All samples were analysed in technical triplicates.

### 2.8 FACS‐based propidium iodide and AnnexinV/7AAD measurement

FACS‐based measurement of cell cycle distribution and apoptosis levels were performed as described previously.^5^, ^6^ All samples were analysed in technical and biological triplicates.

### 2.9 XTT assay

The XTT assay was performed as described previously.^5^, ^6^ Briefly, 24 hours before starting the experiment 5000 cells were seeded in 100 μL standard growth medium per well of a 96‐well plate. The next day, romidepsin or dexamethasone (or both) or corresponding solvents were added to the cells. At the desired time‐points, 50 μL XTT (1 mg/mL) plus 1 μL PMS (1.25 mmol/L) (both from Sigma‐Aldrich) were added and absorbance was measured 4 hours later in an ELISA reader (450 nm).

### 2.10 Chromatin immunoprecipitation followed by sequencing

Data of the chromatin immunoprecipitation followed by sequencing experiment are publically available via GEO (GSE78262) and were re‐analysed in context of this study.^5^

### 2.11 Illumina HT‐12v4 expression and Infinium 450k DNA methylation array

The Illumina expression and DNA methylation array analyses were performed exactly as published.^5^, ^9^ The microarray data sets are available via GEO (ncbi.nlm.nih.gov/geo/) (GSE76709; GSE71239; Data S1E).

### 2.12 Affymetrix expression microarray analysis of GCT tissues

The whole procedure has already been published.^10^ The array was re‐analysed in context of this study.

### 2.13 Statistics

We checked for significance of measured values by performing two‐tailed Student's *t*‐tests. Significance was assumed at *P* ≤0.05. For all measurements, standard deviations were calculated and given above the bars.

## 3 RESULTS

**Figure 1:**
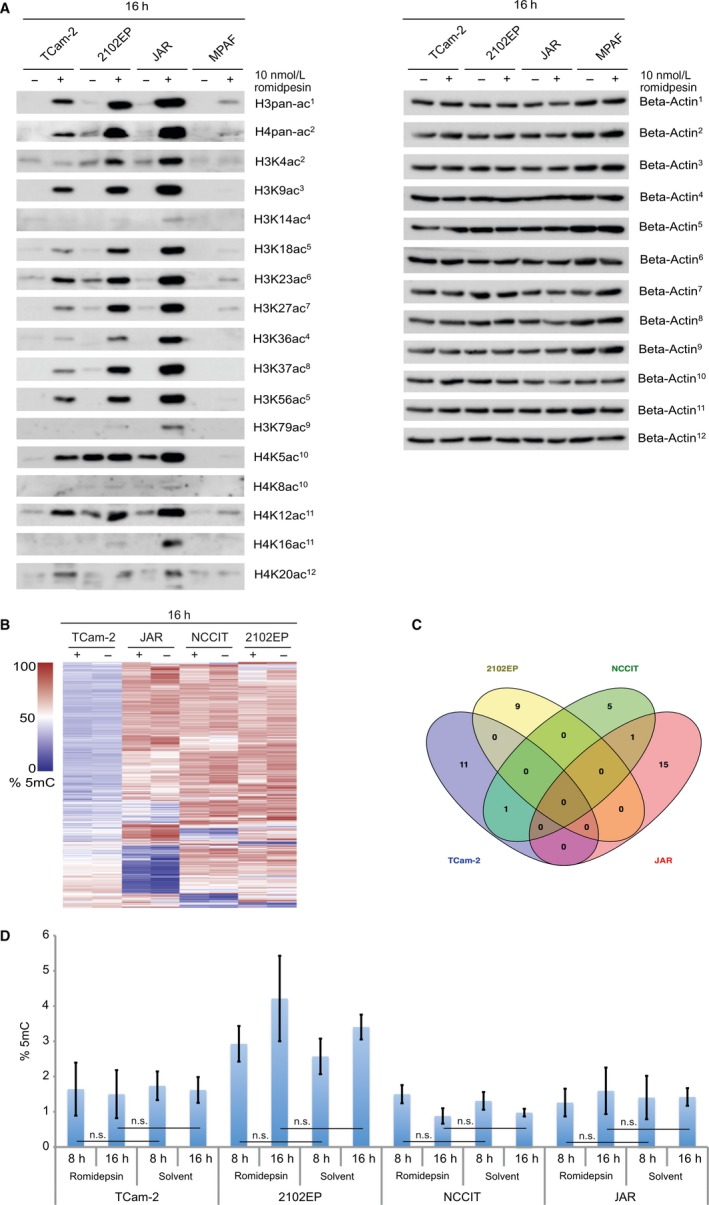
(A) Western blot analysis of lysine acetylation on histones H3 and H4 tails 16 h after 10 nmol/L romidepsin treatment of TCam‐2, 2102EP, JAR and MPAF cells. Beta‐Actin was used as a housekeeper. (B) Heatmap of Illumina 450k DNA methylation microarray data of TCam‐2, 2102EP, NCCIT and JAR cells treated with 10 nmol/L romidepsin (+) or the solvent (−) for 16 h. (C) Venn diagram summarising (common) changes in the DNA methylation landscape of TCam‐2, 2102EP, NCCIT and JAR cells after romidepsin treatment (10 nmol/L romidepsin vs solvent, 16 h). (D) Quantification of 5mC levels in TCam‐2, 2102EP, NCCIT and JAR cells, 8 and 16 h after 10 nmol/L romidepsin or solvent treatment

Previously, we demonstrated that romidepsin causes global hyperacetylation of histones 3 and 4.^5^ Now, we addressed the question, whether romidepsin treatment elicits an alteration at specific lysine residues and acts in a cell‐type specific manner. We used western blotting to screen for changes in lysine acetylation on histones H3 and H4 16 hours after romidepsin application (Figure 1A). General efficacy of the romidepsin treatment was validated by detection of pan‐H3 and ‐H4 acetylation. GCT cell lines (TCam‐2, 2012EP, JAR) showed considerably higher levels of acetylation compared to human fibroblasts (MPAF). Within the group of GCT samples, non‐seminomatous cell lines (2102EP, JAR) showed highest levels of acetylation at all analysed H3‐ and H4‐lysine residues. Four lysine residues (H3K4, H3K14, H3K79, H4K16) showed an increase in acetylation in non‐seminomatous cell lines only. Although, the overall increase in acetylation at these lysines was low compared to the other lysine residues analysed. H4K8 acetylation was low before and remained low after romidepsin treatment in all tested cell lines. No lysine residue could be identified that showed a specific increase in acetylation in TCam‐2 or MPAF cells. Fibroblasts did not respond as strongly as GCT cells to romidepsin, which is in line with the strongly reduced induction of apoptosis in fibroblasts compared to GCT cells.^5^

We analysed if the increase in histone acetylation might also affect the DNA methylation (5‐methylcytosine; 5mC) landscape. It has been proposed that acetylated histones are associated with unmethylated DNA and methyated DNA is able to recruit HDACs to repress transcription.^11^ In addition, in a previous study we demonstrated that GCT cell lines are able to actively demethylate their DNA via the oxidative pathway involving the TET enzymes, allowing for a rapid change in the 5mC pattern.^12^

We performed 5mC microarray analysis (Illumina 450k) and an ELISA‐based quantification of global 5mC levels, but could not detect any significant differences in global 5mC levels of TCam‐2, 2102EP, NCCIT and JAR cells 8 and 16 hours after application of 10 nmol/L romidepsin (Figure 1B,C). Sixteen hours after romidepsin application, 5mC levels of only few genes were altered in GCT cell lines (12 in TCam‐2, 9 in 2102EP, 7 in NCCIT, 16 in JAR; threshold: 50% change in 5mC to control) (Data S1A).

In our previous study, we observed that *DHRS2* was the most prominently up‐regulated gene in response to romidepsin in GCT cell lines, prompting us to analyse the role of *DHRS2* in more detail.^5^ DHRS2 is a NADPH‐dependent dehydrogenase/reductase with 3,4‐hexanedione, 2,3‐heptanedione and 1‐phenyl‐1,2‐propanedione as substrates. DHRS2 has been shown to attenuate MDM2‐mediated P53 degradation, leading to P53 stabilisation and MDM2/P21 accumulation.^13^

First, we demonstrated that *DHRS2* is not expressed in different GCT tissues and normal testis tissues (Figure S1). Furthermore, we demonstrated previously that *DHRS2* is also absent in GCT cell lines and strongly induced upon romidepsin treatment of (cisplatin‐resistant) GCT cell lines, fibroblasts (MPAF and ARZ) and Sertoli cells (FS1).^5^ Thus, up‐regulation of *DHRS2* is a common effect provoked by romidepsin.

Of note, we screened for changes in expression of other *DHRS* genes in response to romidepsin in GCT cell lines, including the important *DHRS2* paralogue *DHRS4* (Figure S1B). We found that, besides *DHRS2*, expression levels of all other *DHRS* molecules analysed remained either unchanged or were down‐regulated upon romidepsin treatment, suggesting that the other *DHRS* genes are not involved in the romidepsin response (Figure S1B).

We asked how *DHRS2* expression might be regulated in GCTs. As shown by chromatin‐immunoprecipitation followed by sequencing in TCam‐2 cells 16 hours after romidepsin treatment, the induction of *DHRS2* was accompanied by an increase in histone H3 acetylation at the *DHRS2* gene locus, suggesting that euchromatin formation might allow for *DHRS2* expression (Figure 3A).^5^

Overall, the DNA methylation levels of four CpG‐dinucleotides across the *DHRS2* gene locus were high (highest levels of 70%‐90% were found at a region of 1500 bp upstream of the transcription start site) and remained nearly unchanged upon romidepsin treatment of GCT cells (Figure 3B). So, DNA methylation of the CpGs analysed is not involved in the up‐regulation of *DHRS2*.

To narrow down the molecular effects of DHRS2 in the romidepsin‐response cascade, we deleted the *DHRS2* locus in TCam‐2 cells using CRISPR/Cas9‐mediated gene editing. By a PCR strategy, we analysed the deletion of the *DHRS2* allele in TCam‐2 cells (Figure S1C,D). In case of wildtype (unaltered) allele, a band of 1200 bp is generated, appearance of a band of 850 bp is indicative of a 3.350 bp deletion within the *DHRS2* coding sequence (Figure S1C,D). We were able to establish three lines heterozygous (TCam‐2‐*DHRS2* ^+/−^) and four lines homozygous (TCam‐2‐*DHRS2* ^−/−^) deficient for the *DHRS2* gene.

A quantitative RT‐PCR (qRT‐PCR) analysis verified that *DHRS2* is not up‐regulated anymore after romidepsin treatment of TCam‐2‐*DHRS2* ^−/−^ cells, while TCam‐2‐*DHRS2* ^+/−^ cells show an induction of *DHRS2* half as strong as wildtype TCam‐2 cells (TCam‐2‐*DHRS2* ^+/+^) (Figure 3C). Interestingly, after romidepsin application partial or complete loss of *DHRS2* had no influence on the expression of the romidepsin key effectors identified in our previous study,^5^ suggesting alternative interaction partners or only a minor role of DHRS2 in the romidepsin cascade (Figure 3D).

Lack of *DHRS2* had no influence on romidepsin‐provoked apoptosis, but caused a significantly lower number of cells to arrest in G2/M‐phase of the cell cycle (Figure 3E,F). These observations are in line with the previous finding that *DHRS2* induction is a common feature of romidepsin treatment, leading to cell cycle arrest in all treated cells (GCT cells, fibroblasts and Sertoli cells), while apoptosis is restricted to GCT cells.^5^ In addition, unaltered apoptosis rates are in line with unchanged expression of stress and apoptosis regulators in TCam‐2‐*DHRS2* ^−/−^ clones compared to TCam‐2‐*DHRS2* ^+/+^ cells in response to romidepsin (Figure 3D,E).

To shed light on the molecular function of DHRS2, we performed expression microarray analysis of the TCam‐2‐*DHRS2* ^−/−^ clones treated with romidepsin or the solvent for 16 hours. The microarray data confirmed that the TCam‐2‐*DHRS2* ^+/+^ cells, in contrast to the TCam‐2‐*DHRS2* ^−/−^ clones, show a strong up‐regulation of *DHRS2* in response to romidepsin (Figure S1E).

We observed that TCam‐2‐*DHRS2* ^−/−^ clones are viable and found that the gene expression profile of TCam‐2‐*DHRS2* ^−/−^ clones and TCam‐2‐*DHRS2* ^+/+^ cells is highly similar without romidepsin application (data S1B), suggesting that DHRS2 is not required for gene regulation and overall survival of GCT cells.

Next, we identified all genes commonly up‐ and down‐regulated in the TCam‐2‐*DHRS2* ^−/−^ clones after romidepsin treatment (vs solvent controls; fold change ≥2) (Data S1C). We compared these gene sets to all genes deregulated in TCam‐2 after romidepsin treatment (Figure S2A,B; Data S1D).^5^ Fifty five genes were specifically up‐regulated in TCam‐2‐*DHRS2* ^−/−^ clones, 70 in TCam‐2‐*DHRS2* ^+/+^ cells (including *DHRS2*) and 127 were commonly up‐regulated after romidepsin treatment (Figure S1A; Data S1D). Among these 127 commonly up‐regulated genes, all of the 23 up‐regulated romidepsin key factors identified in our previous study were found (Data S1D, red‐labelled genes),^5^ confirming the previous qRT‐PCR analysis of the romidepsin key players in TCam‐2‐*DHRS2* ^+/+^, ‐*DHRS2* ^+/−^ *and* ‐*DHRS2* ^−/−^ cells in response to romidepsin (Figure 3D).

Furthermore, we found four genes specifically down‐regulated in TCam‐2‐*DHRS2* ^−/−^ clones, 55 in TCam‐2‐*DHRS2* ^+/+^ cells and 70 commonly down‐regulated genes after romidepsin treatment (Figure S1B; Data S1D). Among the commonly down‐regulated genes, we found three of the four genes identified in our previous study and commonly down‐regulated in GCT cell lines (TCam‐2, 2102EP, NCCIT, JAR) after romidepsin treatment (*NSMAF*,* RCN1*,* ZMYND11*) (Data S1D).^5^ Down‐regulation of *ARID1A* was shown to be the initial step in the romidepsin cascade.^11^ *ARID1A* was also down‐regulated in TCam‐2‐*DHRS2* ^+/+^ and TCam‐2‐*DHRS2* ^−/−^ clones, although in the latter one slightly below the set threshold of fold change ≥2 (−1.83‐fold).

**Figure 2:**
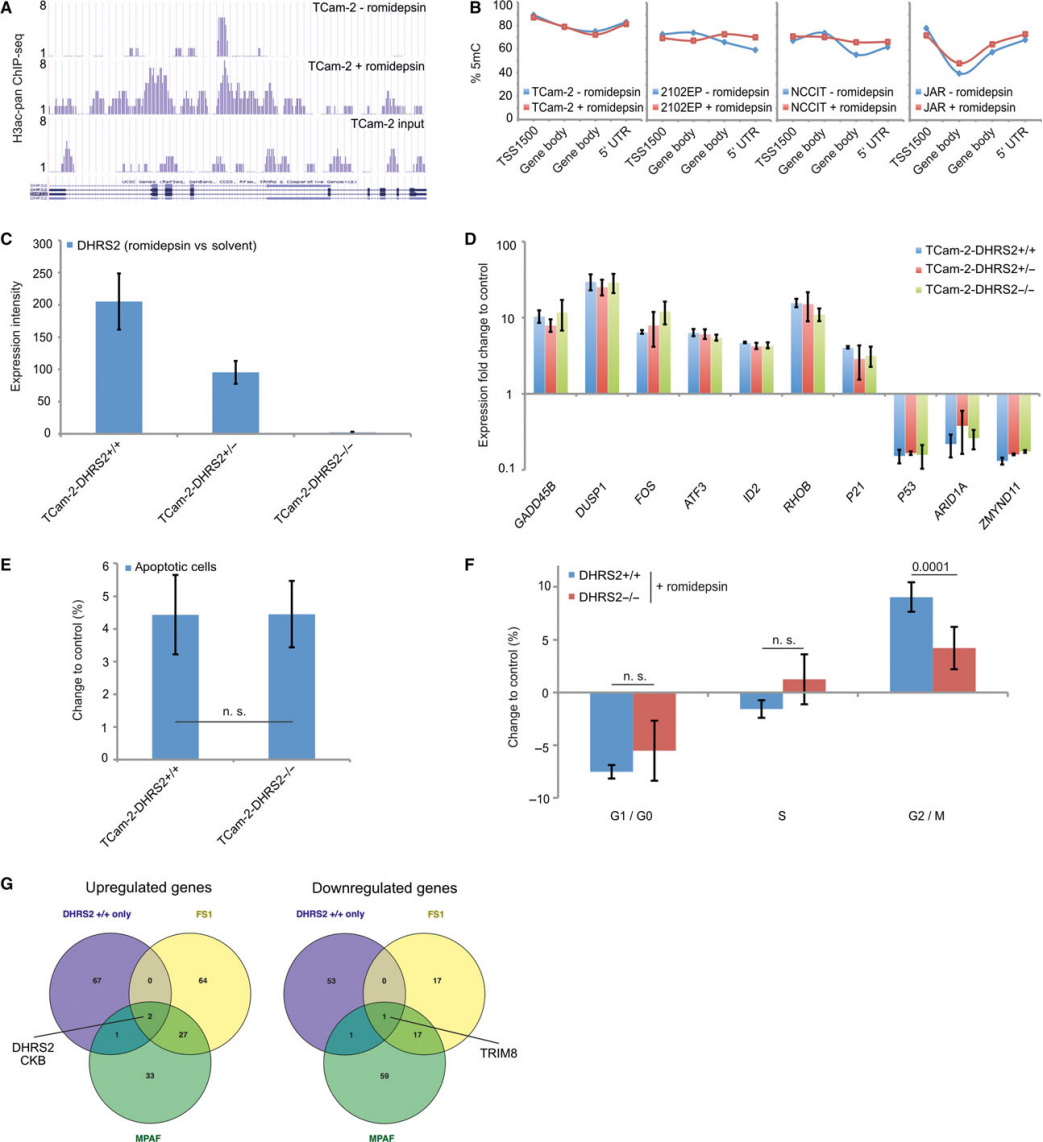
(A) ChIP‐seq data of histone H3 pan‐acetylation levels across the *DHRS2* gene locus of TCam‐2 cells 16 h after 10 nmol/L romidepsin (+ romidepsin) or solvent (− romidepsin) treatment. (B) DNA methylation (5mC) levels across the *DHRS2* gene locus of TCam‐2, 2102EP, NCCIT and JAR cells 16 h after 10 nmol/L romidepsin or solvent treatment. (C) qRT‐PCR analysis of *DHRS2* expression in TCam‐2‐*DHRS2* ^+/+^, TCam‐2‐*DHRS2* ^+/−^ and TCam‐2‐*DHRS2* ^−/−^ clones 16 h after 10 nmol/L romidepsin treatment (vs solvent). (D) qRT‐PCR analysis of the romidepsin key factors in TCam‐2‐*DHRS2* ^+/+^, TCam‐2‐*DHRS2* ^+/−^ and TCam‐2‐*DHRS2* ^−/−^ clones 16 h after 10 nmol/L romidepsin treatment (vs solvent). (E, F) FACS‐based measurement of apoptotic cells (E) and the cell cycle distribution (F) in TCam‐2‐*DHRS2* ^+/+^ and TCam‐2‐*DHRS2* ^−/−^ clones 16 h after treatment with 10 nmol/L romidepsin or the solvent. (G) Venn diagrams summarising the number of genes commonly up‐ or down‐regulated in TCam2‐DHRS2^+/+^ cells, MPAF and FS1 cells (based on microarray data)

We assume that within the group of genes deregulated specifically in TCam‐2‐*DHRS2* ^+/+^ cells after romidepsin application (excluding genes up‐regulated in both, TCam‐2‐*DHRS2* ^+/+^ and TCam‐2‐*DHRS2* ^−/−^ and genes up‐regulated in TCam‐2‐*DHRS2* ^−/−^ cells only) are genes functionally linked to DHRS2 (Data S1A). Furthermore, we theorise that these genes must also be up‐regulated in fibroblasts (MPAF) and Sertoli cells (FS1) after romidepsin treatment, since these cells also up‐regulate *DHRS2* strongly. Thus, we compared all genes up‐regulated or down‐regulated in response to romidepsin in TCam‐2‐*DHRS2* ^+/+^ cells, MPAF and FS1 cells (Figure 2G). We found only two genes commonly up‐regulated, *DHRS2* and *CKB* as well as one gene commonly down‐regulated (*TRIM8*), suggesting a link between *DHRS2* and *CKB* expression/function as well as down‐regulation of *TRIM8*. CKB is a creatine kinase involved in cellular energy homoeostasis including ATP recovery and phosphate transfer between ATP and ADP. TRIM8 (Tripartite Motif‐Containing Protein 8) is a RING‐finger containing protein allowing for protein‐DNA and protein‐protein binding. In addition, TRIM8 is suspected to be an E3 ubiquitin‐protein ligase. In general, this indicates that up‐regulation of *CKB* and down‐regulation of *TRIM8* seem to be related to the strong up‐regulation of *DHRS2* under romidepsin treatment.

**Figure 3:**
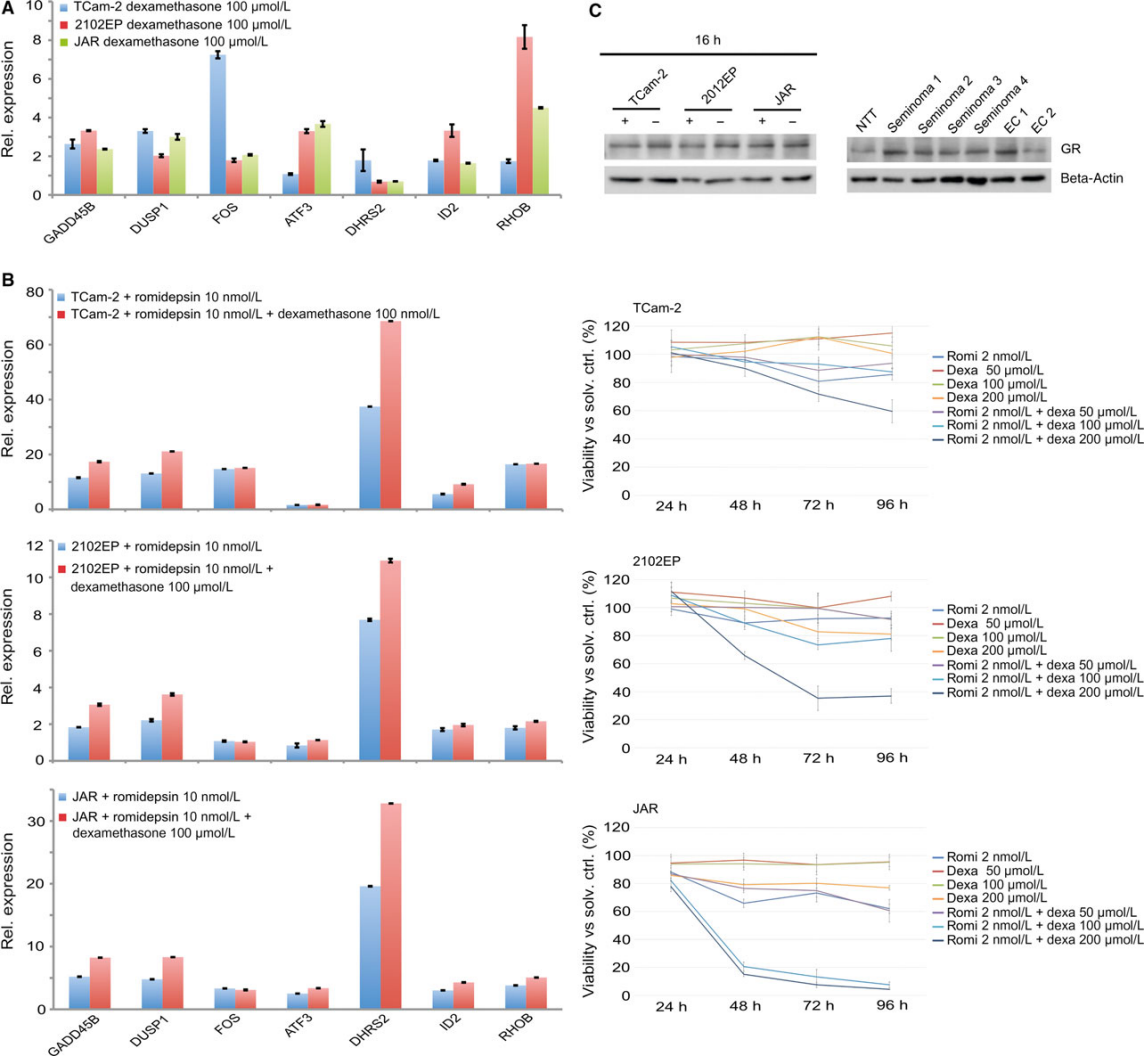
(A) Quantitative RT‐PCR (qRT‐PCR) analysis of indicated genes in TCam‐2, 2102EP and JAR cells treated with 50 μmol/L dexamethasone for 8 days. (B) Left side: qRT‐PCR analysis of indicated markers in TCam‐2, 2102EP and JAR cells after combinatory treatment with 10 nmol/L romidepsin and 100 μmol/L dexamethasone. Right side: XTT assay measuring viability of TCam‐2, 2102EP and JAR cells after individual or combinatory treatment with 2 nmol/L romidepsin and 50/100/200 μmol/L dexamethasone. (C) Western blot analysis of GR expression in GCT cell lines (left side) as well as in seminomas, ECs and normal testis tissue (NTT) (right side) 16 h after 10 nmol/L romidepsin treatment

Glucocorticoids, like cortisol are produced upon stress, reduce nucleosome density and increase H3/H4 acetylation within genomic regions surrounding glucocorticoid‐response elements.^14^, ^15^ In addition, glucocorticoids like dexamethasone are used in the curative treatment of cancer patients and for improving well‐being, physical distress and fatigue.^16^, ^17^ *DHRS2* and other previously identified romidepsin key factors like *GADD45B, DUSP1, FOS, ID2*,* RHOB, ZFP36* and *CRISPLD2* are glucocorticoid responsive genes.^18^, ^19^, ^20^ So, we speculated that a combinatorial treatment with romidepsin and dexamethasone may not only enhance the induction of romidepsin target genes but also counteract therapy‐induced side effects. Treatment of TCam‐2, 2102EP and JAR cells with dexamethasone for 8 days led to up‐regulation of *DHRS2, GADD45B*,* DUSP1*,* FOS*,* ID2, RHOB* and *ATF3* with varying intensities (Figure 3A). In addition, treatment of TCam‐2/2102EP/JAR cells with dexamethasone for 8 days followed by a romidepsin application for 16 hours further boosted up‐regulation of *GADD45B*,* DUSP1* and *DHRS2* (Figure 3B). Expression of *ATF3*,* ID2* and *RHOB* was slightly increased in a cell line‐dependent context, while *FOS* expression was not increased in any cell line analysed (Figure 3B). We asked, if this elevated expression of romidepsin key factors under combinatorial treatment might also lead to an increase in apoptosis rates. Therefore, we treated GCT cell lines TCam‐2, 2102EP and JAR with romidepsin and dexamethasone. In line with these findings, a combinatorial application reduced viability of the tested GCT cell lines more strongly than a single application of romidepsin or dexamethasone (Figure 3B). Here, we used 2 nmol/L of romidepsin, the lowest concentration possible still causing a slow reduction in viability^5^ and dexamethasone concentrations 50, 100 and 200 μmol/L. While the effect of 50 μmol/L was negligible, concentrations of 100 and 200 μmol/L reduced viability in combination with 2 nmol/L romidepsin in a dose‐dependent manner.

Of note, activation of glucocorticoid‐receptor (GR, encoded by *NR3C1*) downstream signalling was linked to HDACi stimulation in human endometrial Ishikawa cells, although expression of GR was not affected.^21^ *NR3C1* is expressed in GCT cell lines and tissues, but not up‐regulated upon romidepsin application, suggesting that romidepsin induces GR signalling downstream targets independent of GR upregulation in GCT cells, too (Figure 3C, Data S1G).

## 4 DISCUSSION

In this study, we further characterised the molecular and epigenetic effects of the HDACi romidepsin on GCT cells.

We demonstrated that romidepsin causes hyperacetylation of the majority of H3/H4 lysine residues across GCT cell lines. Furthermore, non‐seminomas are more sensitive to romidepsin‐provoked changes in histone acetylation than seminomas, indicated by higher levels of acetylation at single lysine residue resolution. In response to romidepsin, we detected acetylation on four lysine residues specifically in EC cell lines (H3K4, H3K14, H3K79, H4K16), which may account for the differences in gene expression between seminomas and non‐seminomas. Fibroblasts presented as least sensitive, reflecting their ability to survive a romidepsin treatment with concentrations suitable to kill GCT cells, presumably by counteracting histone‐wide hyperacetylation.

Furthermore, the strong effects of romidepsin on histone acetylation do not correlate to changes in the DNA methylation landscape (within 16 hours), indicating no crosstalk between both epigenetic mechanisms before induction of apoptosis leads to cell death. Furthermore, these data show that GCT cells do not de novo demethylate their genome in response to romidepsin‐provoked euchromatin formation and thus DNA methylation levels do not contribute to the changes in gene expression detected after romidepsin treatment.

In GCT cells, fibroblasts and Sertoli cells, up‐regulation of *DHRS2* was the most prominent deregulation in gene expression. Previous studies also found a strong up‐regulation of *DHRS2* in various cancer cell lines in response to the HDACi LBH589, vorinostat, SAHA, TSA, MS‐275 and CRA‐024781.^22^, ^23^, ^24^ Thus, up‐regulation of *DHRS2* is a common effect of HDACi treatments. Our results indicate that *DHRS2* up‐regulation seems to be a direct effect of romidepsin‐provoked euchromatin formation and that *DHRS2* has a negligible effect on the expression of romidepsin key factors that mediate stress response and apoptosis, suggesting that up‐regulation of these factors is independent of DHRS2. In line, levels of apoptosis were unchanged between romidepsin treated TCam‐2‐*DHRS2* ^+/+^ and TCam‐2‐*DHRS2* ^−/−^ cells. Interestingly, numbers of cells arrested in G2/M‐phase were lower in TCam‐2‐*DHRS2* ^−/−^ than TCam‐2‐*DHRS2* ^+/+^ cells after romidepsin treatment, indicating a correlation between *DHRS2* up‐regulation and induction of cell cycle arrest. We can exclude that induction of cell cycle arrest is the result of an interaction of *DHRS2* with the romidepsin key players (*ATF3*,* CDKN1A*,* DUSP1*,* FOS*,* GADD45B*,* ID2*,* ZFP36*) since (a) these key factors are not significantly up‐regulated in fibroblasts and Sertoli cells, which also arrest in the cell cycle in response to romidepsin and (b) these key factors are up‐regulated in both, TCam‐2‐*DHRS2* ^+/+^ and ‐*DHRS2* ^−/−^ cells in response to romidepsin.

Hep27 (encoded by *DHSR2*) also has non‐enzymatic activity, that is, a proteolytically processed form of Hep27 can bind the P53‐inhibiting protein MDM2 in the nucleus, leading to accumulation of P53, thereby controlling onset of cell cycle arrest and apoptosis.^13^ Although this mechanism seems plausible in explaining the role of DHRS2 in induction of cell cycle arrest, we found that neither expression nor activity (phosphorylation) of P53 is up‐regulated/induced upon romidepsin stimulus^5^ or *DHRS2* knock out (this study). So, this mode of action of Hep27 can be excluded for romidepsin treated GCTs. Further studies will address the question how DHRS2 is involved in induction of cell cycle arrest under romidepsin treatment.

From our data, we concluded that *DHRS2* up‐regulation is linked to up‐regulation of *CKB*. Thus, romidepsin leads to up‐regulation of two important factors of cellular energy metabolism (DHRS2: NADP/NADPH‐dependent dehydrogenase/reductase; CKB: ADP/ATP‐transfer and binding to ATP‐requiring enzymes). Thus, the strong up‐regulation of both factors under romidepsin treatment might lead to consumption of available cellular energy, contributing to induction of a cell cycle arrest.

In summary, our data suggest that although up‐regulation of *DHRS2* in response to romidepsin is a very prominent effect, *DHRS2* has negligible effect on gene expression and in more detail on the romidepsin key factors. Nevertheless, *DHRS2* can be utilised as a biomarker of a successful romidepsin (HDACi) treatment. Furthermore, these data indicate that alterations in energy metabolism caused by up‐regulation of *DHRS2* and *CKB* might contribute to the cell cycle induction caused by romidepsin.

Finally, we found that the cellular effects provoked by romidepsin in GCT cell lines, like up‐regulation of expression of stress sensors and formation of euchromatin mimic in part glucocorticoid stimulation. A combinatory application of romidepsin and dexamethasone to GCT cells further boosted up‐regulation of romidepsin key factors and efficacy of the romidepsin treatment. A patient might benefit of the combinatorial approach in several ways: (a) the tumour cells might die more quickly, (b) side effects might be reduced/counteracted by dexamethasone and (c) concentrations of romidepsin can be lowered in this combinatorial approach.

## ACKNOWLEDGEMENTS

We kindly thank Anna Pehlke and Blanca Randel for technical assistance. DN and MS are members of UroFors, an association of natural scientists in urology. For further information see: https://www.dgu-forschung.de/wir-ueber-uns/urofors.html.

## DISCLOSURE OF POTENTIAL CONFLICTS OF INTEREST

The authors confirm that there are no conflicts of interest.
