# Supplementary figures and images for: Deciphering the molecular effects of romidepsin on germ cell tumours: DHRS2 is involved in cell cycle arrest but not apoptosis or induction of romidepsin effectors
Source: J Cell Mol Med. 2018 Nov 20;23(1):670–9. doi: 10.1111/jcmm.13971 (PMC6307807; doi:10.1111/jcmm.13971)

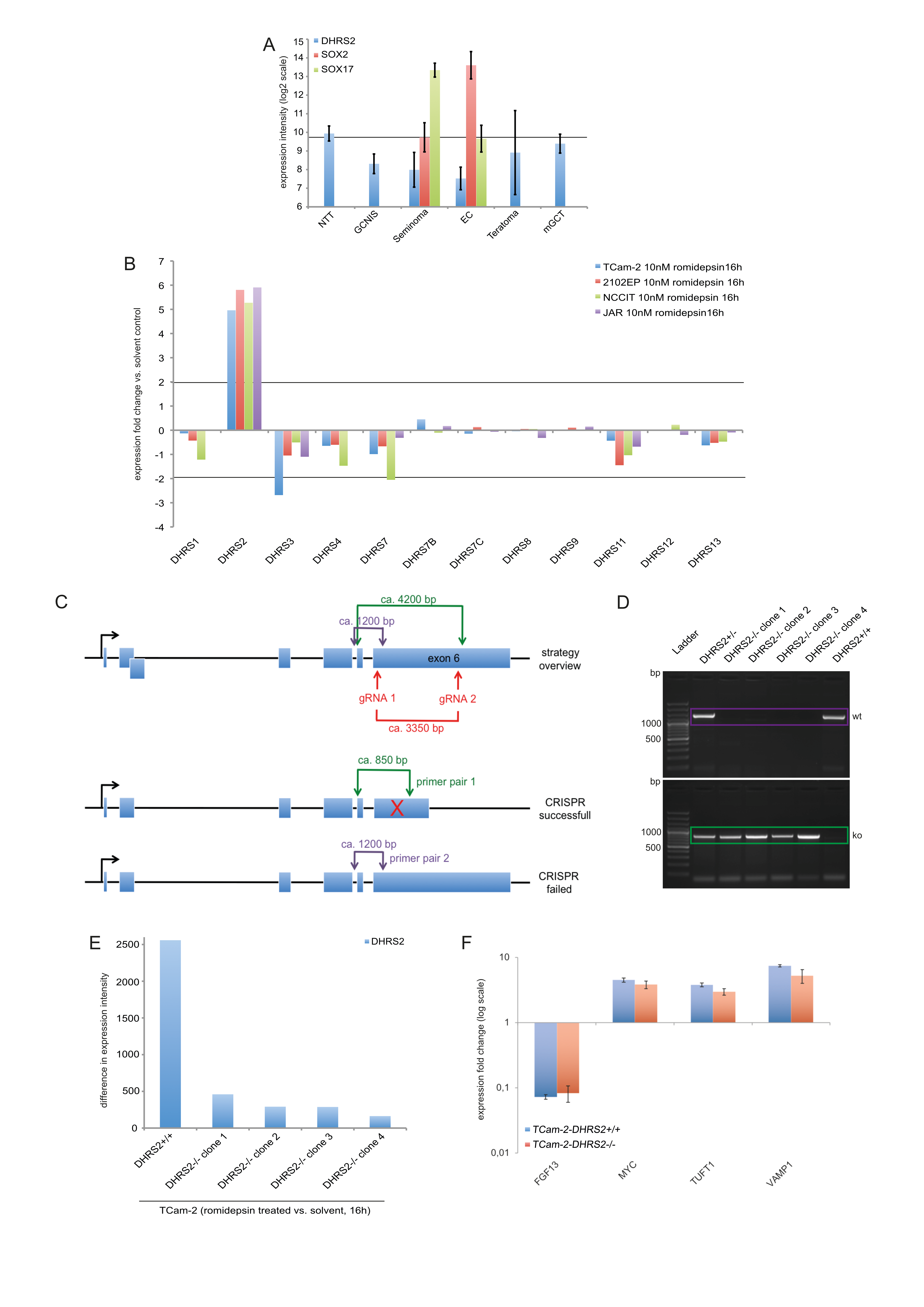

Supplement: Supplementary file 1 [file JCMM-23-670-s001.tif]
